# The Norfolk and Norwich Hospital

**Published:** 1887-09-03

**Authors:** G. Hustler Tuck

**Affiliations:** Chairman of the Board of Management. Salhouse Hall, Norwich


### The Editor's Letter-Box.

[Our Correspondents are reminded that prolixity is a great bar to publication, and that brevity of style and conciseness of statement greatly facilitate early publication.]

### THE NORFOLK AND NORWICH HOSPITAL.

In The Hospital of August 20th it is stated of the Norfolk and Norwich Hospital—"There is, it appears, no regular chaplain, but one of the local clergy receives a grant of £50 a year towards the salary of an additional curate, part of whose function it is to do duty at the hospital." This is incorrect; our chaplain is appointed by the Bishop and the Dean (Law 46); his stipend is £100 a year (annual published report), and the duties belonging to the post are performed with care and efficiency. It is a pity that such a statement should be made without due investigation, and I shall be glad if you will correct it in your next issue.—Yours truly,

G. Hustler Tuck,
Chairman of the Board of Management.

Salhouse Hall, Norwich, August 25, 1887.

*** The statement, which we are glad to find is incorrect, was taken from a letter published in a Norwich paper without comment or contradiction.—[Ed. T. H.]

